# Reflecting on 25 Years of Teaching Animal Law: Is it Time for an International Crime of Animal Ecocide?

**DOI:** 10.1007/s10991-020-09253-0

**Published:** 2020-07-10

**Authors:** Debbie Legge, Simon Brooman

**Affiliations:** 1 The Open University, Milton Keynes, UK; 2 Liverpool John Moores University, Liverpool, UK

**Keywords:** Animal law, Ecocide, Animal welfare, Animal ecocide, Sentience

## Abstract

2019 marked the 25th anniversary of the introduction of Animal Law to the law degree at Liverpool John Moores University. This article examines changes in the legal protection of animals during this time and the impact this will have on research and scholarship in the law relating to animals. We examine whether the overall international treatment of animals has improved and how far the approach to the Animal Law curriculum should be influenced by the growth in concerns around climate change. In this context, we examine the development of the law of ecocide and the extent to which it addresses concerns around animal welfare across the globe. We suggest that those involved in the development of Animal Law, ethics and policy might usefully engage in a new vision of ecocide, which incorporates a clearer notion of ‘animal ecocide’. This new approach would enhance the international and national focus on animals in their own right, would recognise increasing knowledge of animal sentience and would move our responsibilities to them beyond anthropocentric approaches to environmental protection. We argue that the inclusion of a more specific reference to animal ecocide would contribute to the development of Animal Law and would lead to an enhanced relationship between Animal Law and attempts to protect the environment.

## Introduction

### A 25-Year Review of Animal Law

This paper is the result of research undertaken in preparation for a paper delivered to the 2nd United Kingdom Animal Law, Ethics and Policy Conference held in Liverpool in 2019.^1^ In 1994 we introduced a new Animal Law Course onto the law degree at Liverpool John Moores University. It is the most successful such course in the United Kingdom, having been studied by 25–120 final year students each year. The course was originally born out of our concerns for animal welfare in the United Kingdom and beyond. The aim of the 2019 conference paper, and of this resulting article, is to examine how the legal protection of animals has changed since 1997. In particular, have developments to enhance legal protection been successful in protecting more animals or improving their welfare? Have new welfare threats emerged over time? It also led us to consider the context of current concerns for animals alongside those for the environment, in particular, the focus on climate change.^2^ This led us to consider how to create an enhanced link between these strongly related issues.

### The Changing Face of Animal Welfare

The Animal Law course was originally designed around core themes of concern for animal welfare. The socio-legal basis of the course, and its part in the worldwide growth of Animal Law is explored elsewhere (Brooman 2017). However, it is useful to summarise some of the areas of humans’ interaction with animals that are explored in the course, as these have remained largely unchanged:

- The philosophical and ethical implications of human interaction with animals
- Animal experimentation
- Wildlife law and hunting in the UK – with a focus on badgers and foxes
- The international protection of animals
- Biotechnology, animals and the law
- The EU and Animal Welfare
- Animals in Fur Farming
- Animals in Agriculture
- Legal Personhood for animals

Whilst many of the above areas show similar general tends there is insufficient space here to examine changes in each across the life of the course. Therefore, in order to evaluate whether legal protection has been successful here we focus on three examples: animal sentience, developments in relation to whaling and the treatment of wild animals in the United Kingdom.

The philosophical and ethical questions concerning animals have been heavily influenced by developments in our awareness of sentience. In the 1990s, scientific investigations of animal sentience were still emerging but there was already a significant body of evidence that primates, in particular, possess far superior sentient capabilities than had previously been thought. Work on developing language communication skills with primates (e.g. Koko the gorilla and Washoe the chimpanzee), exponentially increased our awareness of sentient capabilities of primates and raised more philosophical questions about our treatment of them.^3^ Contrastingly, during the 1990s these intelligent creatures were the subjects of experiments to, for example, find a cure for Aids. The treatment of higher primates has, arguably improved in that their use in experiments decreased as a result of bans in the United Kingdom (1998) and the European Union 2010.^4^ However, the lower primates are still extensively used in the United Kingdom, and laboratories continue to operate at Oxford University, Edinburgh University, University College London, King’s College London and Cambridge University, even though information on such testing is still largely kept out of the public domain.^5^

Discussions around the implications of our new awareness of animal sentience from science has moved into different areas of welfare concern. For example, there are grave concerns about the ability of animals to feel pain post-stunning in slaughter houses. This kind of concern reflects a notion of ‘sentience’ as the capacity to feel pain, which has been the subject of concern for centuries. This was a concern in the 1990s, and remains so today with the increased use of religious slaughter techniques.^6^

A different version of ‘animal sentience’ has continued to develop in relation to agricultural animals since the Brambell Report (1965). Brambell led to a general improvement in animal welfare by legislating for improvements in husbandry and the general conditions in which farm animals are kept. However, since the 1990s evidence about the farming of wild animals such as mink and foxes has continued to cause concern, as evidence has shown that they are not suitable for close confinement because of their innate ‘wild’ characteristics.^7^ In this context, animal sentience is not catered for as the cages do not provide for the natural and ordinary sentient behaviours that would be part of these animals’ daily lives in the wild. In addition, evidence has emerged about the ‘culling’ of male chicks as part of the egg industry where male chicks are routinely shredded alive in rotating blades.^8^ Concerns have been raised that these animals never experience anything that could amount to a ‘normal’ life and even the simplest exercise of their sentient capabilities. It has led some egg producers in the European Union allowing male chicks a limited life span and has led to calls for a ban on male chick culling in the EU.^9^

The work of Steven Wise, Gary Francione and David Favre,^10^ amongst others, has led to sentience being used as a basis for calling for protecting the personhood of animals. This area has developed significantly since the 1990s, particularly in the United States, leading to court cases and a growing body of support.^11^ Overall, it can be said with some justification that sentience is now one of the cornerstones of claims for the better treatment of animals. It has moved from a primary concern with the sentient capacity to feel pain, to greater claims for the inherent value of animals to experience a natural life, then to claims for the protection of animals in a way that is more congruent with protecting the personhood of human beings.

The second example takes us into the international sphere, with the effectiveness of control of whaling. The moratorium on whaling, introduced in 1986, is considered to be one of the most effective international efforts to protect animals.^12^ Unregulated killing reduced whale numbers to a fraction of that before mass industrialised whaling began. In the early 1980′s television news often showed confrontations between whalers and animal rights activists and the subject was in the public eye. Evidence of slow deaths, combined with evidence of cetacean intelligence led to significant international pressure being placed on whaling nations, including threats to trade.^13^ These welfare concerns and the fear of extinction of some species led to the International Whaling Commission imposing an immediate ban on all but subsistence and scientific whaling in 1986. In the 1990′s and beyond, the leading whaling nations, primarily Norway and Japan, continued to apply pressure to allow managed whaling to recommence arguing that whale numbers had recovered sufficiently, and adding that the moratorium had only been introduced in order to allow numbers to increase sufficiently.^14^

By the time Animal Law was introduced to the legal education curriculum at LJMU in 1994, the debate on whaling had settled into a permanent stand-off between anti-whaling nations and a small number of pro-recommencement antagonists. The IWC, meeting bi-annually, frequently debated recommencement of whaling with growing number of countries supporting this—often under the suspicion that Japan had been offering economic support in return for votes.^15^ However, the moratorium held firm until the 2014 Australia v Japan whaling case in the International Court of Justice.^16^ Australia successfully argued that Japan’s increasing harvest of whales for ‘scientific research’ was both unnecessary and a cover-up for the re-introduction of whale meat into the Japanese markets.^17^ Lacking powers to implement sanctions, the decision was left for Japan to comply. In 2018 Japan announced that it would be leaving the IWC, effectively ignoring the International Court decision, and recommencing industrial whaling, which it did in July 2019. As we write in 2020, Japan’s actions following this decision remain unclear as does any response by anti-whaling nations. There is also evidence that the Japanese public opinion is divided on support for whaling and the chief driver is actually government enthusiasm for the resumption of whaling.^18^ Despite whale meat appearing in the sushi bars of Tokyo its consumption has dropped from 200,000 tonnes a year in the 1960s to 5,000 tonnes.^19^ The overall situation across 25 years remained tense but broadly static in the IWC, with whale numbers increasing. However, the withdrawal of Japan from the IWC, and the apparent recommencement of commercial whaling, is a significant threat to the welfare of whales in the South Atlantic.^20^

The final example is the situation regarding two wild animals in the United Kingdom—foxes and badgers. Concern for the welfare of these animals goes back some time.^21^ Foxhunting has been the subject of intense debate that pitted the Countryside Alliance of farmers, hunters and land-owners against a disparate opposition that sees it as an unnecessary sport with no justifiable benefits.^22^ The election of a Labour government in 1997 committed to legislating to control fox-hunting led to a seven-year battle to determine a legislative formula. The Burns Inquiry^23^ led to the Hunting Act 2004 that marked a significant moment for the hunting of wild animals in the UK. Whilst its basis was clearly the welfare of animals in the chase, the outcome was more of a compromise than might have been anticipated by the anti-hunting lobby—hunting still continues but with severe restrictions on how foxes can be killed. It has led to numerous convictions and so has been lauded by non-governmental agencies such as the RSPCA (Royal Society for the Prevention of Cruelty to Animals), as at least a partial success.^24^ However, the number of convictions (around 350 by 2020) leads to competing claims that the 2004 Act is successful and on the other hand that the number of convictions show contempt for the legislation from the hunting community.^25^ Whether the welfare of foxes has improved is far from clear and the historic debate has moved from the need to legislate to competing calls for the complete abolition or re-design of the legislation.

Legal protection of badgers in the twenty-first century sees a very different picture emerge from that of 1994 when we began teaching Animal Law. At that time the public attention was on the digging of badger setts for the purpose of fighting them with dogs. Public concerns led to the Protection of Badgers Act 1992 that made it illegal, for example, to dig up setts or to cause a dog to enter a sett—it was clearly an Act aimed at preventing a violent form of abuse against badgers. However, the welfare protection of badgers has been severely compromised in the intervening years with the government sanctioned cull.^26^ This highly controversial scheme has led to the licensed destruction of tens of thousands of animals in ethically controversial circumstances. The aim was to reduce the transmission of tuberculosis between badgers and cattle which is claimed to affect the economic viability of cattle farming in the UK—but the evidence of transmission and the effectiveness of the cull have both been heavily criticised. Opponents have vociferously contested the science of the cull and argued that other methods such as vaccination are a moral imperative.^27^ Without making a judgment on the relative merits of the arguments here, it is safe to say that inclusion of a licensing get-out clause in the 1992 Act led eventually to government-sanctioned badger slaughter on a much larger scale than was the case in the 1990s. In March 2020 the government stated that the badger cull would be phased out in favour of vaccination.^28^ However, given the numbers of badgers already culled it is difficult to argue with the contention that the welfare situation for badgers is much worse in the twenty-first century than it was in the last.

To summarise the changing picture of 25 years of Animal Law, and in the context of the best efforts of a large animal welfare community, the success of developing better legal protection of animals has been extremely mixed. Our state of knowledge about animal suffering and awareness is greatly enhanced, but we suggest that this is not reflected in the law. The number of animals killed in ethically dubious practices carried out as a matter of course against them is shocking and shows that ethical arguments for animals have been met with stern opposition, entrenched practices, and government intransigence. The emergence of political/social earthquakes such as Brexit 2016 and the Coronavirus pandemic 2020, along with rising demand for pharmaceuticals, has led to concerns over future trade agreements and the quest for scientific advance using animals taking precedence over concern for animal welfare.^29^ There are some notable exceptions such as the growth in veganism, changes to egg and veal production that show some movement towards better welfare. But this is counterbalanced by concerns over the fate of animals in agriculture, science and the wild. Humans continue to destroy animals claiming it as a natural right or a necessity.

The purpose of the remainder of this article is to discuss whether part of the answer to the question of how best to improve the welfare and standing of animals lies in a greater alignment between the debates over sentience and personhood of animals on the one hand, and concerns over climate change on the other.

### The Proposal for an International Law of Ecocide

Whilst it can be argued, that animal law is a separate discipline to environmental law, the impact of environmental issues on animals cannot be ignored. There is a lot of legislation protecting individual animals from cruelty or harm, or protecting species from extinction, but many of these issues are in fact being caused by environmental harm or the impact of climate change. The loss of large numbers of animals in the Australian bush fires of 2019 is an example.^30^ More recently, residents of some parts of the United Kingdom experienced the loss of pets due to catastrophic floods.^31^

Nikhil Advani, senior program officer for WWF's climate change adaptation programme states:

Conservation biology has traditionally focused on historic threats to species, like habitat destruction and overexploitation. And while addressing those threats remains vital, it's becoming increasingly clear that we need to understand how climate change could harm the various species we’re trying to protect.^32^

Law, in the form of regulations, treaties and domestic legislation provides for a measure of protection of individual species and habitat. However, climate change has brought with it a new set of challenges and has been described as a “super-wicked” policy problem.^33^ This arises from the fact that it is less manageable over time, its effects are seemingly only going to get worse, and it is compounded by the fact that those that are best able to address it are those who primarily cause it. No institution has global jurisdiction or authority to deal with what is a global issue, and solving the crisis remains in the hands of individual governments.^34^ Attempts to deal with this complicated problem are also hampered on a national level by deregulation, voluntary compliance and underfunded regulatory bodies, which can lead to regulatory capture, poor self-reporting and trivialisation of the issues through a lack of effective enforcement action.^35^ It is also exacerbated by the fact that efforts to mitigate or adapt to climate change are seen as “expensive, unnecessary, futile and remote from policies that yield immediate and politically popular economic benefits”.^36^

There are also general problems with climate change litigation, such as justiciability in relation to standing and the separation of powers. There is the question of whether the courts are the appropriate bodies to hear and resolve questions of equity, rights and obligations in relation to climate change or whether it is a political issue for the legislature. Much of the litigation is based on constitutional provisions,^37^ or can piggy-back onto human rights issues (right to health/life/ freedom of conscience)^38^ refugee law,^39^ statutes or common law. If an issue cannot be dealt with under these headings, courts can be wary of expanding their remit and are accused of overstepping their powers by, effectively, legislating.^40^

Despite these limitations, there has been a significant amount of climate change litigation. The Climate Change database for 2019 records 294 cases against governments and 29 against companies worldwide, with a further 723 in the USA, often based on the doctrine of public trust. Litigation remains an important tool to push policy makers and market participants to develop effective means of climate change mitigation.^41^ However, despite the growth in climate change litigation the fact remains that there are limits to the effectiveness of the legal control of climate change. Much of the law is reactive, taking place after damage is caused and when it is difficult to remedy, as in the Niger Oil case.^42^ This case saw Shell accepting responsibility for significant environmental damage, but the tragedy was that it could, and should, have been avoided. National civil law based actions such as those in negligence or nuisance do not prohibit, prevent or pre-empt damage and have the built-in limitation of reaching only common law jurisdictions.^43^ The conflict of interest that arises from the challenge of climate change means that national law is inadequate to deal with the global nature of the crisis.

In light of these limitations and gaps in extant provision, a conclusion was set out by the late Polly Higgins that we need to turn elsewhere—to international criminal law and a law on ecocide. When the lack of progress on animal welfare is also considered, we conclude that this should be accompanied by the addition of animal ecocide into this emerging new doctrine.

### Issues in Defining and Implementing a Crime of ‘Ecocide’

In 2010, the late Polly Higgins^44^ proposed an amendment to the Rome Statute^45^ of the International Criminal Court (1998)^46^ to include a crime of ecocide in order to address the anomaly that, outside periods of war, it is not a crime to cause mass destruction or loss of ecosystems.^47^ In the proposed amendment to the Rome Statute, ecocide would become a new Crime Against Peace. Higgins, Short and South define ecocide as:

…the extensive damage to, destruction or loss of ecosystem(s) of a given territory, whether by human agency or by other causes to such an extent that peaceful enjoyment by the inhabitants of that territory has been or will be severely diminished.^48^

This built on the work undertaken by Mark Allen Gray,^49^ and there is an ongoing campaign to include the crime of ecocide in international law. The prosecution of a crime of ecocide has been tested in a mock trial in the UK Supreme Court to test how it would work in practice.^50^ At the ICC 2019 annual assembly, three pacific islands and the campaign for ecocide held a panel event on the official assembly programme discussing the role of the ICC in prosecuting ecocide.^51^ The arguments have been set out elsewhere^52^ and so just a brief outline of the law will be provided here.

The first argument for an international crime of ecocide is it removes the enforcement of environmental law from the domestic arena. An example can be seen in the outwardly positive development that ten countries have introduced a law on ecocide although this advance has been undermined by the fact that many of these countries suffer from corruption.^53^ Higgins et al. suggest that an addition to the international criminal code would facilitate the potential effectiveness of international law in preventing crimes against the environment.^54^ Another advantage Higgins et al. set out is the ability to take pre-emptive action via an international criminal law of ecocide that is preventative, moving us from a principal of the polluter paying to one where the polluter does not pollute at all.^55^

When such a change is viewed alongside other types of enforcement such as those mentioned above and the soft law of other International agreements such as the Paris Agreement,^56^ those pushing for a crime of ecocide highlight the potential for a greater impetus for changing behaviour. It contains the added ingredient of being based on restorative justice, providing for the restoration of what has been lost rather than applying a punishment to the perpetrator.^57^ In effect, a law of ecocide would help prevent and prohibit human-based damage, and could take greater account of natural catastrophes. The route to bringing a case would be through national courts and/or the International Criminal Court, and this would provide a legal duty of care so opening the possibility of holding governments and corporations to account.^58^

The choice of whether to protect habitats, the environment more widely or species is also affected by emerging conversations centred on rights. Arguments for attributing rights to animals or granting them personhood have a long history,^59^ but there have been more recent collateral moves towards ascribing rights to nature.^60^ In addition to legal personhood becoming a large area of research in Animal Law^61^ and Environmental Law, and a significant backdrop to a law of ecocide,^62^ there is also discussion around the notion of creating a specific area known as Wild Law^63^ under the ambit of Earth Jurisprudence.^64^ The proposal for a law of ecocide recognises many of the notions intrinsic to these new ways of thinking about protecting the environment through law—that we are all inhabitants of the earth and that our interconnectedness with nature is central to understanding how we can survive. These conversations mark a significant increase in the output of legal scholars into discussions about law’s relationship with the holistic ecosystem, and sits within the ideas of Gaia theory,^65^ the web of life^66^ and ecocentrism.^67^ It seems that the legal academy has realised the need to contribute to discussion about how law can help reverse the damage of anthropocentrism which, it is argued, has caused a radical discontinuity between human and other forms of nature/being.^68^ Ecocide joins these new conversations as a way in which humans could develop a greater connection between law and issues such as climate change. It helps us to find new ways of thinking about our existence and that of other species such that we can begin to solve issues rather than merely punish perpetrators, as well as providing a practical route by which this could be achieved. It leads us to question the role of law in determining and defining a new relationship between humans, animals and the environment.

A change in the law to incorporate an international law of ecocide would provide not just moral but legally enforceable norms to address the balance whereby law tends to focus on human interests over the natural world.^69^ This issue is discussed by Capra and Mattei, who argue that it is the system of law itself that is the problem as it focuses on private ownership, capital and state sovereignty and that what is needed is a law of ecology based on social and natural relations and community ownership.^70^ But most importantly a wider notion of ecocide helps us debate issues outside of narrow confines, and revealing the interconnectedness of the law and issues such as climate change. It can help us take a wider view of issues such as the effect of having pets, of veganism, of the loss of top soil, or of farming on the wider environment, which need new thinking and new action in order to be able to solve the issues. Ecocide may also help us debate new issues and challenges such as gene modification and the farming of insects as food.

In the context of a changing climate there are no simple solutions to the moral dilemmas arising from our relationship with animals. Criminal actions might go through a number of routes, some of which might not necessarily be tackled through an action based on harm to the animal. Farm animals, for example, have a significant impact on climate change through their emissions^71^ and the welfare of such animals is also of serious concern. However, it could be argued that the industry might be best held to account through the damage it causes to the environment with these emissions and the loss of habitats when land is cleared for livestock production.^72^ Environmental damage is highly visible and of immediate concern to those who live with it.

We accept that the practicalities of introducing this new international crime leave some issues to be resolved. Anastasia Greene supports the need to address ‘the massive problem of environmental destruction’,^73^ but suggests that the definition proposed by Polly Higgins is too broad and does not sit comfortably within the current ambit of the International Criminal Court, because of its lack of environmental expertise. She suggests that a new forum, a specialised international court, may be needed. Despite these doubts, there is a great deal of momentum behind the need to create greater accountability for environmental destruction which continues to attract considerable support. The proposal would give greater weight to enforcement rather than seeing states continue to water down the ineffective diplomatic efforts to prevent long-lasting devastation.^74^

### The Case for Including a specific crime of Animal Ecocide

What place might the protection of animals have in this new law of ecocide? Some of the prime movers of this new doctrine have mentioned that harm to animals should be included in the realm of potential actions. Higgins et al. suggest that examples of environmental harms and crime should include crimes/harm against animals/non-human species, such as: ‘abuse, mistreatment or death of animals and birds’ by war, catastrophe, oil spills, deforestation, medical experiments, farming, clearance of land for development, water and air pollution, soil erosion, climate change, wildlife trafficking and scientific advances.^75^ This highlights a weakness as far as animals are concerned, as whilst animals are mentioned in the argument, the protection afforded to animals is tied up in the language of protecting the environment and human interests:

‘Abuse, mistreatment or death of animals and birds may be visible and stark as in cases of destruction of habitats by war, catastrophe, oil-spills, deforestation, or be less visible and socially accepted when related to farming, medical experiments, clearance of land for building, or where damage results from activities that cause air or water pollution, soil-erosion or climate change.’ (Higgins et al 2013 253).

We agree with the sentiments of this definition of ecocide, in that such a connection with environmental damage is often very much associated with collateral damage to the environment, however we suggest that tying the fate of wild animals so closely to environmental concerns mask issues that relate to animals alone in light of their sentient capacities.

A definition which gave more prominence to animal sentience would accord with notions of personhood that have become highly defined in the twenty-first century. It would protect animals in their own right, not by reason of their being members of a species, and it would recognise their right to live life according to their species and give them freedom from unnecessary pain. We suggest a definition as follows, which draws on the definition of ecocide that was presented by UK lawyer Polly Higgins to the United Nations Law Commission in 2010:

Animal Ecocide is the unnecessary killing or slaughter of a wild or wild-caught animal, by any human agency, or allowing such killing or slaughter to be so caused by any governmental organisation, to such an extent that an animal, or group of animals, lose their sentient capacity to live a natural life according to their species.

Just as with claims in the area of environmental ecocide it would be possible for international animal protection organisations to bring actions for crimes against those species leading to the build-up of precedent in the area regarding definitions of ‘unnecessary’ etc. This would allow new and existing scientific evidence to be brought to court and a legal standard of proof to be applied to what often passes as acceptable behaviour by government decree.

In this claim to draw on the definition of ecocide to develop one for animal ecocide we would argue there should be a distinction between wild and kept animals. Animals kept for farming, experimentation, pets and entertainment are already covered by legislation that, whilst it needs to be strengthened, e.g. by greater penalties for abuse or restrictions on their use in experiments, does provide a measure of protection. It also avoids dampening potential action by re-heating arguments around the moral case for eating animals where the meat industry becomes extremely defensive and argues necessity.^76^ This article suggests that it is in the area of wild animal welfare that the call for a crime of animal ecocide would significantly advance the welfare for animals and not get caught up in matters of domestic control/existing provision. The international surge in concern over climate change and destruction of the environment could provide an opportunity for those calling for different attitudes to other species and/or better welfare for animals to join voices. We suggest that this could be done by modifying calls for an international law of ecocide to include a clearly identifiable definition of animal ecocide, which might provide a collateral advantage of creating a stronger claim for the law of ecocide more generally.

Animals are sentient creatures with lives that exist beyond their usefulness to humankind or any connection with the environment. Indeed, some issues that we contend would be covered by a specific animal-centred definition of ecocide would not necessarily be covered by the current definition of ecocide. An example of this is whaling, where the suffering of animals is paramount beyond the potential environmental impact of the loss of species. We draw on the arguments put forward for the personhood of animals to assert that just as humans are protected against unnecessary pain not because of their membership of a species, so it should be now for some animals such as the primates, whales and dolphins. These highly sentient creatures should be accorded a similar right to life and freedom from, for example, torture or oppression. We suggest that a definition of animal ecocide would outlaw all forms of whaling beyond those for subsistence of some humans in the far-reaches of cold areas of the planet.^77^ In the vast majority of cases, the killing of whales, porpoises and dolphins would amount to animal ecocide—it is a practice that has no reasonable scientific justification and involves the unnecessary infliction of pain on highly sentient creatures where alternative sources of science and/or food are available. This would also help to highlight the impact fishing, ship strikes and plastic pollution^78^ are having on whales, leading to loss of life.^79^

Such a definition of animal ecocide could also give rise to potential actions against governments for allowing the unnecessary suffering of wild domestic species. An example is the highly contentious cull of badgers in the United Kingdom. This sort of activity might be covered by the existing definition of ecocide—but it would certainly be covered by a new definition of animal ecocide. The case would come down to the competing claims of farmers/government—i.e. that this is a necessity using the best scientific methods available where no other reasonable alternative to protect other interests can be found, against those who argue that the cull is based upon dubious science and the unnecessary and inadequately controlled suffering of a wild species where clear alternative course of action are available. It could also be used to take a government to court where its legislation was considered to be inadequate to control specific animals such as the United Kingdom’s Hunting Act of 2004 (foxes and deer). Outside the UK it could be used to prevent, for example, the mass destruction of wild birds in particular those who by virtue of their migration routes are truly international species.

## Conclusion

Conducting a review of Animal Law after 25 years of teaching presented us with some stark realities—the prospects for animal welfare at both national and international levels, remains precarious. We feel that this situation might be improved by building on the proposed law of ecocide to more specifically recognise animal ecocide.

As we move forward, climate change is arguably the most important factor in the protection of animals because of the potential harm caused through the destruction of ecosystems. Damage to the environment and assaults on the welfare of animals often go hand in hand, as illustrated by the examples of bush fires in Australia and flooding in the United Kingdom mentioned above. The interconnectivity of animal harm and environmental damage is further complicated by the fact that pet ownership itself is blamed for creating environmental damage through emissions, which in turn adds to the potential for flooding and fire.^80^

As a subject for legal education, the course has had a significant impact on individuals as evidenced in very positive feedback. It evidences that students become more aware of the environmental and ethical issues raised by humans’ interactions with animals. For example:

‘I enjoyed learning about the different topics and laws surrounding animal welfare. Also, how relevant the topics are today and how the change in animal welfare may also have an impact on the environment, for example faux fur.’ 3^rd^ year student 2020.’

‘Exploring this module was very interesting. It highlights extremely important issues.’ 3^rd^ year student 2019.

Students are evidently interested and concerned by the issues raised in studying Animal Law and most often align with arguments raised by the animal welfare and environmental communities. As conversations move onto the impact of human activity on the planet, the links with Earth Jurisprudence, Wild Law and Environmental Law will all assume much greater prominence in legal scholarship due to the issues raised by the Covid19 pandemic, apparently caused by faulty interaction with wild animals, disappearing species and universally-held concerns over climate change.^81^ It is accepted that our behaviour needs to change, and it follows that laws to govern human interaction with the natural world will do likewise. We suggest that the more specific inclusion of animal ecocide in existing proposals for an international crime of ecocide adds to its coherence by recognising the need to protect the creatures that live in the wild.

We recognise that the proposal for an international law of ecocide has some distance to travel to be workable in practice and to find its appropriate home.^82^ However, we suggest that in developing such claims there needs to be a greater connection between the movements for animals and those for the environment. This could be enhanced by creating an international law of ecocide that more clearly recognises the importance of animal species. The specific definitions and practicalities of ecocide may be in their infancy, but this does not make them less important for the future, on the contrary, we recognise that important movements for change have often started with uncertainty and moved gradually to acceptance. At one time there was no notion of Environmental Law, nor of Animal Law, now there are courses, laws, conferences, numerous organisations and governmental agencies to deal with both. As Mark Allen Gray said of a proposed international crime of ecocide in 1996: ‘Criminalization will occur because it must.’ It is important that those who describe themselves as ‘animal lawyers’ join the conversation about the criminalisation of practices that inflict damage on the planet and its creatures, as well as damaging the environment for humans. We have explained here one way in which we think scholars of animal law can join the conversation.

We suggest that our proposed definition of animal ecocide clarifies the argument for an international crime of ecocide as it recognises the importance of wild species and avoids environmental criminal codes being viewed through purely anthropocentric eyes. It expands the basis of ecocide beyond human rights.^83^ It would contribute to persuading international and national law and policy makers to make the necessary legal changes to ensure both a sustainable future and the protection of species in their own right. Our review led us to conclude that legal scholars’ concerns for animals, and concerns for the environment, are two sides of the same coin. Establishing an international code of environmental and animal ecocide would help to bridge the gap, and so assist with the protection of all animal species including our own.

## Footnotes

1 A short video summary can be seen at https://www.youtube.com/watch?v=a9Tdof6QuVM&t=93s

2 A preliminary paper on this subject was delivered to the Conference on Animal Law, Ethics and Policy 2019 hosted by Liverpool John Moores School of Law in conjunction with the United Kingdom Centre for Animal Law. The section on ecocide also formed part of a discussion paper for the Strathclyde Centre for Environmental Law and Governance at Strathclyde Law School, 2019.

3 Rumbaugh and Washburn (2008).

4 Chatfield and Morton (2018).

5 Linzey and Linzey (2017).

6 Brooman (2016).

7 Pickett and Harris (2015).

8 See RSPCA report at https://kb.rspca.org.au/knowledge-base/what-happens-with-male-chicks-in-the-egg-industry/; Fioranelli et al. (2019).

9 https://globalnews.ca/news/6420754/male-chick-culling/

10 Wise (1995); Francione and Charlton (2017) and; Favre (2017).

11 Wise (2018).

12 Hurd (2012).

13 Anable (1993).

14 Kojima (2019).

15 Butler-Stroud (2016).

16 Whaling in the Antarctic (Australia v. Japan: New Zealand Intervening), International Court of Justice (31 March 2014), available at https://www.icj-cij.org/en/case/148.

17 Telesetsky et al. (2014).

18 Butler-Stroud (2016).

19 McCurry, J, *The Guardian* 1st July 2019.

20 Wilkinson (2020).

21 Cassidy (2019).

22 Parry (2019).

23 Burns et al. (2000).

24 RSPCA (2015).

25 RSPCA (2020).

26 McCulloch and Reiss (2017).

27 McCulloch and Reiss (2017).

28 Carrington (2020).

29 Sparks and Brooman (2017).

30 Zhou (2019)

31 BBC News 17/02/2020.

32 Advani (2015).

33 Lazarus, (2009).

34 UN (2017) 7–8.

35 UN (2017) 7–8.

36 UN (2017) 7–8.

37 Chrisafis (2020).

38 Urgenda Foundation v The State of the Netherlands (Ministry of Infrastructure and the Environment) Case C/09/456,689/HA ZA 13–1396 (English translation) See the case commentary by Pocklington D, Environmental Law and Management/2015 - Volume 27/Issue 2, 1 October/Case Notes/Climate change and human rights–the Urgenda case – (2015) 27(2) ELM: 61.

39 Lyons (2020). https://tbinternet.ohchr.org/_layouts/15/treatybodyexternal/Download.aspx?symbolno=CCPR%2fC%2f127%2fD%2f2728%2f2016&Lang=en.

40 UN (2017) 27–39.

41 https://climatecasechart.com/.

42 Konne (2014).

43 UN (2017) 34–6.

44 Higgins (2012) 61.

45 The Rome Statute is the governing document for the International Criminal Court and covers crimes against peace. These are “the most serious crimes of concern to the international community as a whole”, over and above all other laws. It covers crimes such as genocide, crimes against humanity, war crimes and crimes of aggression. https://www.icc-cpi.int/resourcelibrary/official-journal/rome-statute.aspxhttps://legal.un.org/icc/statute/romefra.htm.

46 UN General Assembly, *Rome Statute of the International Criminal Court (last amended 2010)*, 17 July 1998, ISBN No. 92-9227-227-6, available at: https://www.refworld.org/docid/3ae6b3a84.html [accessed 14 May 2020].

47 Higgins et al. (2013).

48 Higgins et al. (2013) 257.

49 Gray (1996).

50 https://ecocidelaw.com/the-law/mock-trial/, https://sites.google.com/site/349924e64e68f035/issue-4/ecocide-mock-trial-case-comment.

51 https://www.stopecocide.earth/.

52 Higgins et al. (2013).

53 Grechenkova (2017).

54 Higgins et al. (2013) 263–4.

55 Higgins et al. (2012).

56 https://unfccc.int/process-and-meetings/the-paris-agreement/the-paris-agreement.

57 Rivers (2012).

58 Higgins et al. (2013) 264.

59 Brooman and Legge (1997) chapters 1 and 3.

60 Eckstein et al. 2019.

61 Giménez-Candela Derecho (2018) 5–28.

62 Naffine (2012) 68–83.

63 Burdon (2010); White, (2014).

64 Cullinan (2010) and (2011).

65 Lovelock (2000).

66 Capra (1996).

67 Kopnina et al. (2018) 140–148.

68 Kopnina et al. (2018).

69 Higgins et al. (2013) 256.

70 Capra and Mattei (2015) 197.

71 Grant (2019).

72 Cameron and Cameron (2017).

73 Greene (2018) 47.

74 Lay et al. (2015)

75 Higgins et al. (2013) 253–4.

76 Despite this pragmatic step, we maintain our commitment to the moral case for veganism.

77 We do not condone such use of whales for subsistence but recognise that this is a difficult decision weighing up human and animal rights where the current situation has no easy resolution. Such extreme difficulties of resolving conflicting human and animal interests do not occur in other examples that would be covered by Animal Ecocide.

78 Ellenby (2019).

79 Bergman (2020).

80 Hickman (2019).

81 See *The Guardian’s* Environment page for coverage of all these issues. https://www.theguardian.com/uk/environment.

82 Greene (2018).

83 Greene (2018).

## References

[CR1] Advani, N. 2015. Animals Affected by climate change, https://www.worldwildlife.org/magazine/issues/fall-2015/articles/animals-affected-by-climate-change. Accessed 3rd June 2020.

[CR2] Anable K (1993). NAMMCO defies the international whaling commission's ban on commercial whaling: are whales in danger once again. Transnational Law.

[CR3] Bergman, M. 2020. We’re watching them die: can right whales pull back from the brink? *The Guardian* 17 April 2020. https://www.theguardian.com/environment/2020/apr/17/north-atlantic-right-whales-were-watching-them-die. Accessed 20th May 2020.

[CR4] Brambell FWR (1965). Report of the technical committee to enquire into the welfare of animals kept under intensive livestock husbandry systems: (Command Rep. 2836).

[CR5] Brooman S, Legge D (1997). Law relating to animals.

[CR6] Brooman S (2016). In Search of the missing ingredient: religious slaughter, incremental failure and the quest for the right to know. Journal of Animal Ethics.

[CR7] Brooman S (2017). Creatures, the academic lawyer and a socio-legal approach; introducing animal law into the legal education curriculum. Liverpool Law Review.

[CR8] Burdon P (2010). Wild law: the philosophy of earth jurisprudence. Alternative Law Journal.

[CR9] Burns L, Edwards V, Marsh J, Soulsby L, Winter M (2000). Committee of Inquiry into hunting with dogs in England and Wales.

[CR10] Butler-Stroud C (2016). What drives japanese whaling policy?. Frontiers in Marine Science.

[CR11] Cameron, J. and S. Cameron. Animal agriculture is choking the Earth and making us sick. We ust act now. *The Guardian,* 4th December 2017. https://www.theguardian.com/commentisfree/2017/dec/04/animal-agriculture-choking-earth-making-sick-climate-food-environmental-impact-james-cameron-suzy-amis-cameron. Accessed 1^st^ June 2020.

[CR12] Capra F (1996). The web of life: a new scientific understanding of living systems.

[CR13] Capra F, Mattei U (2015). The ecology of law: toward a legal system in tune with nature and community.

[CR14] Carrington D. 2020. `Siesmic Shift’: Ministers signal end of Badger Cull, *The Guardian*, 5 March 2020. https://www.theguardian.com/environment/2020/mar/05/badger-cull-phased-out-replaced-vaccinations-bovine-tb-england. Accessed 3rd June 2020.

[CR15] Cassidy A (2019). Protecting the Badger? *Vermin, Victims and Disease*.

[CR16] Chatfield K, Morton D, Schroeder D, Cook J, Hirsch F, Fenet S, Muthuswamy V (2018). The Use of Non-human Primates in Research. Ethics Dumping.

[CR17] Chrisafis, A. 2020. French NGOs and local authorities take court action against Total, *The Guardian*, 27th January 2020. https://www.theguardian.com/world/2020/jan/27/french-ngos-and-local-authorities-take-court-action-against-total. Accessed 3rd June 2020.

[CR18] Cullinan, C. 2010. Earth jurisprudence: from colonization to participation. *State of the World*, 143–148.

[CR19] Cullinan C (2011). Wild law: a manifesto for earth justice.

[CR20] Eckstein G, D’Andrea A, Marshall V, O’Donnell E, Talbot-Jones J, Curran D, O’Bryan K (2019). Conferring legal personality on the world’s rivers: A brief intellectual assessment. Water International.

[CR21] Ellenby, D. 2019. The five: Species affected by plastic pollution, *The Guardian,* 4 August 2019, https://www.theguardian.com/environment/2019/aug/04/five-species-affected-by-plastic-pollution. Accessed 30th May 2020.

[CR22] Favre D, Kalof Linda (2017). Animals as living property. The Oxford Handbook of Animal Studies.

[CR23] Fioranelli M, Sepehri A, Roccia MG, Rossi C, Petar Vojvodic P, Lotti J, Barygina V, Vojvodic A, Wollina U, Tirant M, Thuong NV (2019). In Ovo Sexing of Chicken Eggs by Virus Spectroscopy. Open Access Macedonian Journal of Medical Sciences.

[CR24] Francione GL, Charlton AE, Kalof Linda (2017). Animal rights. The Oxford handbook of animal studies.

[CR25] Giménez-Candela T (2018). Dignity, sentience, personality: the legal relationship between animals and humans. Derecho Animal.

[CR26] Grant, H. 2019. Global food Producers - failing to face up to role’ in climate crisis, The Guardian, 4 September 2019, https://www.theguardian.com/environment/2019/sep/04/global-food-producers-climate-crisis. Accessed 2nd June 2020.

[CR27] Gray M (1996). The international crime of ecocide. California Western International Law Journal.

[CR28] Greene A (2018). The campaign to make ecocide an international crime: quixotic quest or moral imperative. Fordham Environmental Law Review.

[CR29] Grechenkova OY (2017). Certain problems of fighting ecocide. Journal of Advanced Research in Law and Economics (JARLE).

[CR30] Hickman, L. 2019. Britain's problem with pets: they're bad for the plane, *The Guardian*, 13 November 2009. https://www.theguardian.com/environment/2009/nov/13/ethical-living-carbon-emissions. Accessed 3rd April 2020.

[CR31] Higgins P (2012). Eradicating ecocide.

[CR32] Higgins P, Short D, South N (2012). Protecting the planet after Rio–the need for a crime of ecocide: Polly Higgins, Damien Short and Nigel South propose a way forward to deal with climate change and environmental deterioration. Criminal Justice Matters.

[CR33] Higgins P, Short D, South N (2013). Protecting the planet: a proposal for a law of ecocide. Crime, Law and Social Change.

[CR34] Hurd I (2012). Almost saving whales: the ambiguity of success at the International Whaling Commission. Ethics & International Affairs.

[CR35] Konne BR (2014). Inadequate monitoring and enforcement in the Nigerian oil industry: the case of Shell and Ogoniland. Cornell International Law Journal.

[CR36] Kopnina H, Washington H, Taylor B, Piccolo JJ (2018). Anthropocentrism: more than just a misunderstood problem. Journal of Agricultural and Environmental Ethics.

[CR37] Kojima C (2019). Japan: Japan’s decision to withdraw from the international convention for the regulation of whaling. Asia-Pacific Journal of Ocean Law and Policy.

[CR38] Lay B, Neyret L, Short D, Baumgartner MU, Oposa AA (2015). Timely and necessary: ecocide law as urgent and emerging. Journal Jurisprudence.

[CR39] Lazarus RJ (2009). Super wicked problems and climate change: Restraining the present to liberate the future. Cornell Law Review.

[CR41] Linzey A, Linzey C (2017). The ethical case against animal experiments.

[CR42] Lovelock J (2000). Gaia: a new look at life on earth.

[CR43] Lyons, K. 2020. climate-refugees-cant-be-returned-home-says-landmark-un-human-rights-ruling, *The Guardian*, 20 January 2020. https://www.theguardian.com/world/2020/jan/20/climate-refugees-cant-be-returned-home-says-landmark-un-human-rights-ruling. Accessed 5th May 2020.

[CR44] McCulloch S, Reiss M (2017). Bovine tuberculosis and badger culling in England: an animal rights-based analysis of policy options. Journal of Agricultural and Environmental Ethics.

[CR45] Naffine N (2012). Legal personality and the natural world: on the persistence of the human measure of value. Journal of Human Rights and the Environment.

[CR46] Parry L (2019). Discourses on foxhunting in the public sphere: a Q methodological study. British Politics..

[CR47] Pickett, H., and S. Harris. 2015. The case against factory fur farming: A scientific review of animal welfare standards and" WelFur." A report for Respect for Animals.

[CR48] Rivers L (2012). Shareholder return - a Nuremberg defence - Ecocide and Restorative Justice. Environmental Law and Management.

[CR49] RSPCA, 2015. https://news.rspca.org.uk/2015/07/13/the-current-hunting-act-works-says-rspca-after-three-hunters-prosecuted-for-illegal-activity/

[CR50] RSPCA, 2020. https://www.rspca.org.uk/getinvolved/campaign/hunting/facts

[CR51] Rumbaugh D, Washburn D (2008). Intelligence of apes and other rational beings.

[CR52] Sparks P, Brooman S (2017). Brexit: a new dawn for animals used in research, or a threat to the ‘most stringent regulatory system in the world’? a report on the development of a brexit manifesto for animals used in science. UK Journal of Animal Law.

[CR53] Telesetsky A, Anton D, Koivurova T (2014). ICJ's decision in *Australia v. Japan:* giving up the spear or refining the scientific design?. Ocean Development & International Law.

[CR54] United Nations. 2017. Report: Burger, M., Gundlach, J., Kreilhuber, A., Ognibene, L., Kariuki, A., & Gachie, A. 2017. The status of climate change litigation. A global review. New York, NY: United Nations Environment Programme. https://wedocs.unep.org/bitstream/handle/20.500.11822/20767/climate-change-litigation.pdf?sequence=1&isAllowed=y. Accessed 6 July 2020.

[CR55] White, S. 2014. Wild Law and animal law. In *Wild Law-In Practice*, ed. M. Maloney and P. Burdon, 247. Routledge.

[CR56] Wilkinson DM (2020). The use of domestic measures to enforce international whaling agreements: a critical perspective. Denver Journal of International Law & Policy.

[CR57] Wise SM (1995). The legal thinghood of nonhuman animals. Boston College Environmental Affairs Law Review.

[CR58] Wise SM (2018). The struggle for the legal rights of nonhuman animals begins-the experience of the nonhuman rights project in New York and connecticut. Animal L..

[CR59] Zhou, N. 2019. Koala fact-check Australian bushfires survival species at stake. *The Guardian,* 26 November 2019. https://www.theguardian.com/environment/2019/nov/26/koala-factcheck-australian-bushfires-survival-species-at-stake, Accessed 12th May 2020.

